# Feasibility of implementing a rapid-learning methodology to inform radiotherapy treatments: key professional stakeholders’ views

**DOI:** 10.1136/bmjonc-2023-000226

**Published:** 2024-03-13

**Authors:** Arbaz Kapadi, Gareth Price, Corinne Faivre-Finn, Rebecca Holley, Kate Wicks, Kathryn Banfill, Gareth Webster, Kevin Franks, Fiona McDonald, Daniel Johnson, David P French

**Affiliations:** 1 Manchester Centre for Health Psychology, Division of Psychology and Mental Health, School of Health Sciences, The University of Manchester, Manchester, UK; 2 Division of Cancer Sciences, School of Medical Sciences, The University of Manchester, Manchester, UK; 3 The Christie NHS Foundation Trust, Manchester, UK; 4 Worcestershire Oncology Centre, Worcestershire Acute Hospitals NHS Trust, Worcester, UK; 5 Leeds Cancer Centre, St James's Institute of Oncology, Leeds Teaching Hospitals NHS Trust, Leeds, UK; 6 The Royal Marsden NHS Foundation Trust, London, UK; 7 James Cook Cancer Institute, South Tees Hospitals NHS Foundation Trust, Middlesbrough, UK

**Keywords:** Radiotherapy, Radiation oncology

## Abstract

**Objective:**

Pragmatic methodologies, often termed rapid-learning, are being pursued that can match the pace of innovation in radiotherapy and generate evidence from the real-world treatment setting. It is important to understand the feasibility of implementing such pragmatic approaches before their application in practice. This study investigated key professional stakeholders’ perceptions and opinions of rapid-learning and real-world data (RWD).

**Methods and analysis:**

Twenty-three interviews were conducted with key professional stakeholders based across five UK radiotherapy cancer centres. Centres varied in size and reflected different healthcare environments. Data were collected between December 2022 and May 2023, and analysed using inductive thematic analysis.

**Results:**

Four themes were generated: (1) the alignment of rapid-learning methodologies with the reality of practice, (2) concerns related to the variability of RWD, (3) the maturity of data infrastructures and capacity for rapid-learning and (4) further support, education and evidence needed to convince stakeholders to adopt rapid-learning approaches.

**Conclusion:**

The potential of rapid-learning to help address evidence gaps in radiotherapy development was positively received by different professional stakeholders. However, the effectiveness of rapid-learning was viewed as being highly dependent on the collection of quality data in the routine setting, while the variable set-up at different cancer centres is also likely to be a key challenge for potential implementation. Developing data infrastructures to improve data interoperability was considered crucial for rapid-learning implementation, along with method clarity, educational support and training for radiotherapy teams.

**WHAT IS ALREADY KNOWN ON THIS TOPIC**

- There is significant interest in applying pragmatic rapid-learning approaches that use real-world data (RWD) to evaluate technique and treatment changes introduced in oncology practice. It has been suggested that rapid-learning may help provide evidence that is timelier and more representative than evidence generated from traditional clinical trials.

**WHAT THIS STUDY ADDS**

- There are few investigations regarding the feasibility and acceptability of rapid-learning approaches and RWD to different professionals involved in radiotherapy. This study provides insight into the views of key professional stakeholders towards the potential strengths and challenges of rapid-learning implementation across UK cancer centres.

**HOW THIS STUDY MIGHT AFFECT RESEARCH, PRACTICE OR POLICY**

- The findings from this study suggest how implementation of rapid-learning is likely to require a multifaceted approach, underpinned by strong and clear organisational support. Further research to outline critical data points and address method safety may be needed along with investment into creating suitable conditions for implementation, which notably includes development of data infrastructures and provision of support for professionals.

## Introduction

There has been considerable evolution in radiotherapy over the last few decades with multiple different technical changes implemented in treatment workflows. The transformation in radiotherapy over this period has mainly occurred through successive incremental changes rather than revolutionary step changes.^1 2^ Evaluating the clinical and cost-effectiveness of these incremental changes, however, can be challenging.^3–5^ There are several experimental methodologies that may be used to evaluate the effect of interventions in radiotherapy with their respective strengths and limitations well described in the literature.^1 3 6^ Traditionally, evidence from conventional randomised controlled trials (RCTs) has been used to evaluate changes in clinical practice.^1 6 7^ RCTs are considered to have high internal validity, through which cause and effect relationships between interventions and outcomes can be established.^6–9^ However, RCTs can be resource-intensive, may confer limited generalisability and their results can take time to implement in clinical practice.^10–14^ Failing to formally evaluate the impact of technical changes can result in the adoption of treatments that are less effective or produce more adverse effects than previous practices.^3^

In seeking to improve practice more quickly than RCT evidence allows, alternative pragmatic methodologies are being pursued.^15–18^ One such approach is to generate evidence from the real-world treatment setting using real-world data (RWD) and to track changes through iterative cycles of ‘rapid-learning’.^18 19^ The rapid-learning approach is informed by the model for improvement which provides a structured framework for using continuous learning cycles to test, evaluate and build learning from small scale changes.^20 21^ Rapid-learning in cancer treatment development proposes a similar approach, using RWD to evaluate the impact of changes between learning cycles.^15 16 18^ The National Institute for Health and Care Excellence defines RWD as ‘data relating to patient health or experience or care delivery collected outside the context of a highly controlled clinical trial’.^22^ These data are routinely collected as standard of care about all patients, for example, information collated in patients’ electronic health records (EHRs), data derived from product or disease registries or data from other sources that can inform health status.^23^ It has been suggested that the diversity of RWD may help generate evidence that is more representative than evidence generated from traditional clinical trials.^24–26^ However, RWD may not always be structured, which can make it difficult to aggregate and analyse.^24 27^

**Figure 1:**
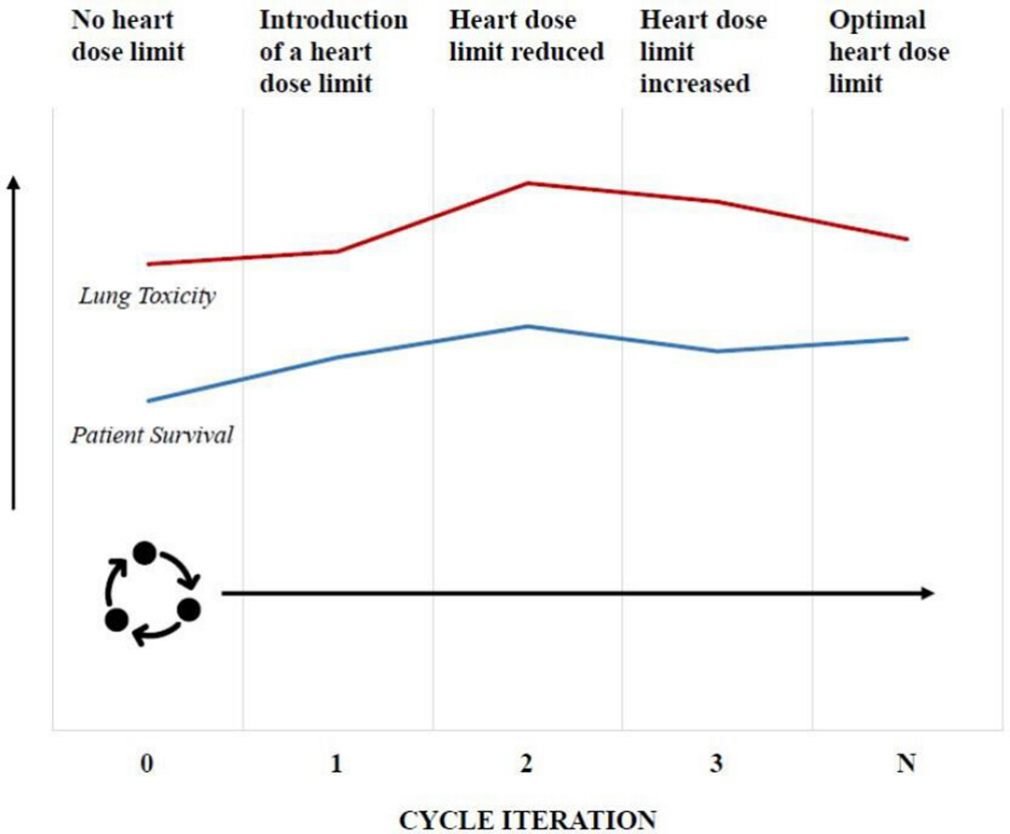
Conceptual illustration of how the rapid-learning approach will be used to find the optimal heart dose limit during thoracic radiotherapy treatment. Starting with no heart dose limit, baseline survival and lung toxicity is assessed using retrospective data. A heart dose limit is introduced in cycle 1 resulting in increased survival and slightly increased lung toxicity. If toxicity levels are acceptable the dose limit is decreased further in cycle 2, resulting in unacceptable lung toxicity risk. The dose limits are then raised to reduce toxicity in cycle 3. Further cycle iteration will take place where heart dose limit is increased and decreased until the balance of risks is considered clinically optimal.

While concepts of rapid-learning and RWD have been well discussed in the literature, there are few investigations of its use in clinic.^1 9 28 29^ The RAPID-RT programme seeks to demonstrate the clinical effectiveness of rapid-learning to the radiotherapy setting.^18 30^ The programme is built around a clinical implementation of the approach to evaluate the impact on patient outcomes of changing thoracic treatment to limit heart dose (box 1 and figure 1).^18 30^

Box 1 **RAPID-RT study - clinical exemplar**

Recent evidence shows that irradiating the top of the heart while treating lung cancer increases the risk of premature death.^38 53–57^ The specific anatomical area includes the ascending aorta, right atrium and right coronary artery. It is postulated that the conduction system may be damaged directly by radiation or indirectly through inflammation, fibrosis or ischaemia; dose to the superior vena cava and left atrium have been associated with ECG changes.^58 59^ There is, therefore, an emerging consensus that heart dose should be reduced during radiotherapy.

In response to the strength of the evidence, heart-sparing radiotherapy, where a dose limit has been included for this cardiac avoidance region, has been introduced as a new standard of care at The Christie National Health Service (NHS) Foundation Trust for all stages I–III non-small cell lung cancer patients treated with non-stereotactic ablative body radiotherapy. The RAPID-RT study^18^ aims to use only RWD to provide evidence of the impact of this change in practice on patients’ clinical outcomes. The aim is to regularly update the clinical team with analytical results showing observed changes in outcome and thereby offer the opportunity to refine the new dose limit in successive learning cycles to maximise patient benefit.

The primary clinical end points of the RAPID-RT study are changes in 12-month overall survival (expected to improve by 10%–20%) and acute (within 4 months of radiotherapy) treatment related toxicities, in particular, incidence of grade 3+ radiation pneumonitis and oesophagitis. The clinical study will also be used as a vehicle to evaluate the opportunities and barriers to establishing rapid-learning as a new NHS evaluation framework for technical changes in radiotherapy.

Alongside demonstrating clinical effectiveness, it is critical to evaluate wider questions regarding the ethical acceptability and feasibility of rapid-learning. Repurposing routinely collected health data, for example, requires important ethical consideration in areas such as data security, ownership and compliance with legal provisions.^31 32^ Establishing public trust in rapid-learning is also crucial given there will be a change to the treatment patients receive.^15^ The permissibility of using RWD in rapid-learning forms a separate programme of research within the RAPID-RT study.^18^ Similarly, exploring the acceptability of rapid-learning to professionals is important as rapid-learning is based on an evidence base that has traditionally not informed intervention development; this forms the focus of this article.

### Study aim

In this article, we report on the findings from a study that aimed to explore the feasibility and acceptability of implementing rapid-learning in the clinic. The study examines key professional stakeholders’ views towards the potential strengths and challenges of implementing rapid-learning in practice.

## Methods

### Design and setting

This study adopted a multicentre, qualitative interview-based design. Five UK cancer centres participated in this study: The Christie National Health Service (NHS) Foundation Trust (Manchester) (host study site), The Royal Marsden NHS Foundation Trust (London), Leeds Cancer Centre (St James’s Institute of Oncology, Leeds Teaching Hospitals NHS Trust, Leeds), Worcestershire Oncology Centre (Worcestershire Acute Hospitals NHS Trust, Worcester) and James Cook Cancer Institute (South Tees Hospitals NHS Foundation Trust, Middlesbrough). These sites were chosen as (a) they were geographically diverse, (b) they reflected different healthcare environments (varying in size and academic capacity) and (c) they served populations with varying demographic characteristics. Data collection took place between December 2022 and May 2023.

### Participants and procedure

Participants were eligible for the study if they were a professional based across the following areas: (1) clinical oncology, (2) radiotherapy physics and treatment planning, (3) digital learning, informatics and innovation, and (4) research and information governance. Gathering these different perspectives was important as introducing a change in radiotherapy requires cross-department collaboration. Purposive sampling was used with participants identified through consulting with clinical service leads at respective sites. After identification, individual study invites and participant information sheets (PIS) were circulated via email to participants by the RAPID-RT study team (AK) asking whether they wished to participate in a one-off interview.

In total, 23 participants were interviewed (table 1). For several reasons, a large number of these interviews took place with personnel based at The Christie (Manchester). This is the first site at which implementation of rapid-learning in lung cancer radiotherapy takes place and we further anticipated that professionals may be, to some extent, more familiar or knowledgeable about rapid-learning. Subsequently, this sample represented ‘information-rich’ cases with informed views that we intended to capture.^33^ All participants were knowledgeable about the types of data that may be grouped under RWD. Knowledge of the rapid-learning concept and methodology varied; knowledge appeared greatest among clinical professionals (clinical oncologists, medical physicists and treatment planning) based in the large cancer centres. None of the participants interviewed were members of the immediate RAPID-RT study team. Data collection continued until the research team were satisfied that sufficient data had been collected and no new information was being generated. In this manner, our study sample size was informed by the concept of information power^34^—the more relevant information a sample holds, the fewer participants are needed.

**Table 1:** Setting and participants

Site		Description	No of interviews	Participant roles
A	The Christie NHS Foundation Trust (Manchester) (Host study site)	Large academic centre	13	Clinical oncologists: seven Physicists/clinical scientists: four Dosimetrist: one Radiographers: two Treatment planning: two Digital learning and data informatics: two Medical directive team: two Clinical outcomes directive team: one Research and information governance: two
B	The Royal Marsden NHS Foundation Trust (London)	Large academic centre	3	
C	Leeds Cancer Centre, St James’s Institute of Oncology, Leeds Teaching Hospitals NHS Trust (Leeds)	Large academic centre	2	
D	James Cook Cancer Institute, South Tees Hospitals NHS Foundation Trust (Middlesbrough)	Medium-sized acute trust	2	
E	Worcestershire Acute Hospitals NHS Trust (Worcester)	Small non-academic centre	3	

NHS, National Health Service.

### Interviews and topic guide

Semi-structured interviews were conducted virtually (over MS teams or Zoom) and ranged between 35 and 60 min; the median interview time was 45 min. Semi-structured interviews were considered an effective method to explore participants’ perceptions of rapid-learning while encouraging further questions to emerge from the discussion itself.

Two original interview guides were developed between members of the RAPID-RT research team comprising a range of expertise that includes qualitative methods, implementation science, health psychology, radiotherapy and oncology. While sharing several questions, the first guide (designed for clinic-based professionals) involved a slightly greater focus on understanding clinical evidence, whereas the second guide (designed for management, data informatics and research personnel) entailed more focus on data and governance-related areas. Key areas such as data handling, governance and infrastructure, patient facing workflows and operational capacity were covered across interview guides (online supplemental file 1). For purposes of making abstract ideas more concrete for participants less familiar with the ideas being discussed, a clinical exemplar was attached to the PIS as a pre-interview reading exercise for all participants (figure 1). In the interview, this RAPID-RT clinical exemplar was introduced in more detail by the interviewer (AK) to help stimulate further discussion. The timing of introducing the exemplar during interviews differed based on familiarity shown by participants towards rapid-learning.

10.1136/bmjonc-2023-000226.supp1 Supplementary data

### Analysis

The majority of interviews were audiorecorded and transcribed verbatim by an independent professional transcription team. One participant did not consent for their interview to be recorded and notes were taken instead. Data were analysed in NVivo V.12 using thematic analysis.^35^ Thematic analysis enables the identification of representative patterns of participants’ views and experiences from across the dataset. Each transcript was systematically read multiple times for familiarisation prior to coding by AK. Coding was conducted at an inductive-manifest level, whereby codes were derived from the data rather than being guided by preconceived ideas. Coding was iterative with developing codes compared and refined across transcripts. Patterns were identified within the codes and initial themes were created by AK. Themes and codes were compared across the dataset with analysis concluded when no new codes were generated from the data. Codes, developing themes and the final thematic structure were reviewed and refined by AK, DPF, GP and CF-F (online supplemental file 2).

10.1136/bmjonc-2023-000226.supp2 Supplementary data

## Results

Coding was organised under four themes: (1) the alignment of rapid-learning methodology with the reality of practice, (2) concerns related to the variability of RWD, (3) the maturity of data infrastructures and capacity for rapid-learning and (4) further support, education and evidence needed to convince stakeholders to adopt rapid-learning approaches. Participant quotes illustrating themes can be found in table 2.

**Table 2:** Illustrative quotations from participants

Theme 1: The alignment of rapid-learning methodology with the reality of practice		Theme 2: Concerns related to the variability of RWD	
Description	Captures participants’ views on the current evidence landscape, and how rapid-learning may positively address evidence gaps and complement practice.	Description	Captures participants’ views regarding the quality of RWD and how this could pose a challenge to rapid-learning implementation.
Subtheme(s)	Quotations	Subtheme(s)	Quotations
Challenges of RCT evidence and the need for complementary methodologies	‘The purist RCT just isn’t a feasible model because it takes too long and it’s often not real-world because it’s selected patients. There is still a place for RCTs, but we need an alternative model to drive improvements through data and evidence.’ *The Christie (Manchester*)	Scepticism over the accuracy and robustness of RWD	*Incomplete/missing data* ‘When you look at RWD, you find loads of problems, you will find lack of data, missing data, data not recorded very well, and it’s quite hard to work out what was going on. That’s the reality, because it’s not always recorded brilliantly, because people are just busy, that’s the problem.’ *Leeds Cancer Centre (Leeds)* *Poor follow-up toxicity data* ‘It’s the gaps in the data. Are we pulling all the data together from across the entire pathways to be able to answer that question fully? And with toxicity data, we will often see the patient once or twice after radiotherapy and then refer them back to the local team for continued follow-up, and we're not routinely collecting any data there. I don't think we're going to have a comprehensive data set on our practice at the minute, acute effects yes, but not late effects.’ *The Christie (Manchester)*
Rapid-learning may provide timely evidence	‘Lots of benefits if you can do this (rapid-learning) properly. It can be timelier, there’s a continuing sort of feedback loop, so you're continuing to optimise those outcomes from RWD. There’s always some selection bias within the RCT process and RWD should be able to overcome that.’ *The Christie (Manchester*)		
Rapid-learning may offer potential for greater treatment personalisation	‘The thing that we’d like to be doing, that we’re not, is individualising our treatments (…) we just have no information other than maybe the clinician has a hunch. So if we could turn that hunch into something a bit more reliable, that is backed up with RWD, that would be good.’ *Worcestershire Oncology Centre (Worcester*)		
Incremental changes to radiotherapy are routinely implemented with ‘less evidence’	‘We make changes on an evidence base far less than this (rapid-learning and RWD). At least you can say the methodology is novel, and that you’ve got a plan. We’ll often make changes based on very little evidence so I’m not worried about people saying I don’t like this methodology.’ *Leeds Cancer Centre (Leeds*)		

Theme 3: The maturity of data infrastructures and capacity for rapid-learning		Theme 4: Further support, education and evidence needed to convince stakeholders to adopt rapid-learning approaches	
Description	Captures participant reflection on how to address identified challenges regarding data quality and capacity of centres to implement rapid-learning.	Description	Captures participants’ views towards what they considered may be needed to inform further acceptability of rapid-learning implementation across radiotherapy teams.
Subtheme(s)	Quotations	Subtheme(s)	Quotations
Integration of clinical and digital services	‘You’ve got to have all of those things, clinical and digital aligned for a better data strategy, and the trouble is, the clinicians are so deflated by their experience with digital yet we still ask them to do more with little support and that brings a whole load of sighs and mistrust.’ *The Christie (Manchester*)	Study methodology clarification	*Addressing safety and mitigating risk* ‘It’s trying to come down to understand what the tolerances are. What is a risk level that’s acceptable? And going through I suppose the existing processes within a healthcare system and clarify: ‘these are our controls around that, this is how we deal with that’. That stuff will be important for us to know about (for the effectiveness of rapid-learning).’ *The Royal Marsden (London)* *Timeliness of cycles* ‘The cyclical approach only works if you either have the toxicity developing fairly soon after the change, or there is a bio-marker for toxicity that you can measure which can predict outcomes. For example, to measure cardiac toxicity, you’re waiting for somebody to have a cardiac event but you can be waiting five, ten years for that. Does that mean there’s five to ten years between each cycle?’ *The Christie (Manchester*)
Capacity of different centres to implement rapid-learning	‘We need to try and make sure that these discussions (about rapid-learning and RWD) are relevant to smaller centres because there is the ivory tower concept. It’s critical to try and get buy-in from them (smaller centres). The problem is the more you go out to the small centres, then you start to have resource issues in terms of how they can support this and do they also have the digital infrastructure for this work.’ *Leeds Cancer Centre (Leeds*)	Buy-in from professionals and cancer centres	*Analytical support/time and space* ‘We need accessibility and visibility of analytical personnel. You need both to work together because you can put the clinical perspective in from what teams think they can see in the data and vice versa, but then it’s about having the time and the space to have that dialogue.’ *James Cook Cancer Institute (Middlesbrough)* *Confidence in rapid-learning* ‘If you want good confidence in it (rapid-learning), you need a good data set, you need some semblance of a surveillance policy - how will it be safe and quality assured? - and then you need a methodology that supports patient care, otherwise it will not have the buy-in. That support and clarification will increase people’s confidence.’ *The Royal Marsden (London*)

RCT, randomised controlled trial; RWD, real-world data.

### Theme 1: the alignment of rapid-learning methodology with the reality of practice

This first theme captures participants’ views on the current evidence landscape, and how rapid-learning may positively address evidence gaps and complement practice. RCT evidence was described as gold standard and robust, with participants noting high internal validity (ability to determine cause-and-effect relationships) as a particular strength. This said, overwhelmingly, all participants discussed RCTs in response to the challenges experienced in practice. Along with time, finance and resource costs of preparing and conducting RCTs, participants highlighted strict eligibility criteria, often applied in trial recruitment and resulting in the participation of certain patient groups (‘self-selected,’ ‘age-specific’ patients), as a barrier to generalising trial outcomes to the real-world population.

To address some of the identified challenges of RCTs, participants reflected on how rapid-learning could provide a complementary methodology. Participants, for example, viewed access to routinely collected RWD and the cyclical design of rapid-learning as an opportunity to generate evidence within a more reasonable time frame than currently experienced. Moreover, clinical oncologists and treatment planning personnel discussed how rapid-learning and RWD could offer potential to provide more personalised radiotherapy treatment; this was by virtue of RWD containing information about diverse patient communities. Personalisation of treatment was highlighted as an ongoing development within radiotherapy and represented a particular ambition for all centres. Participants described how personalisation not only offered potential for greater treatment choice but could also help optimise resources and minimise wastage.

Clinic-based professionals, across centres, recalled various experiences of making incremental changes to radiotherapy techniques and practices that were not informed by ‘highest quality’ RCT evidence. Situations where such incremental changes were introduced included changing dose limits and applying organ-at-risk constraints, responding to uncommon tumours and diagnoses, and delivering radiotherapy through uncertain service periods, for example, the COVID-19 pandemic. Exploring the rationale behind introducing incremental changes to techniques, participants highlighted several factors that included clinical protocols and guidelines, clinician experience, local consensus among teams and auditing practices. Clinical audit appeared to be customary practice across all centres. Participants, who were less familiar with rapid-learning, likened the cyclical approach in rapid-learning to their clinical audit practices on review of the clinical exemplar; the use of RWD to evaluate outcomes represented the major difference. Subsequently, rapid-learning was viewed not too dissimilar from existing practices currently employed and potentially offered a way to ‘formalise’ processes already taking place such as introducing incremental changes.

### Theme 2: concerns related to the variability of RWD

This second theme captures participants’ views regarding the quality of RWD and how this could present a challenge to rapid-learning implementation. All participants raised some scepticism over the accuracy and robustness of clinician-reported and patient-reported datasets as they drew on their respective experiences. Participants described how datasets can often be incomplete and missing data, while data may not always be recorded systematically (eg, use of different data recording formats) making it difficult to analyse. The collection of clinician-reported data, for example, was described on several occasions by participants as a burden with limited time and resource for healthcare staff. In discussion around toxicity data, vital to evaluating outcomes in rapid-learning, the informativeness of this data could be impacted by the failure to collect robust follow-up data. This concern was expressed by staff across all centres which they placed on losing contact with individuals after their discharge.

The completeness of patient-reported outcome measure (PROM) data was also raised. Some doubts were cast over the accuracy of PROM data, with some clinicians noting how, in their view, this type of data was not always well documented, for example, under-reporting or over-reporting of symptoms. Equally, issues regarding accessibility (eg, ability for certain groups to provide this data and IT literacy) were raised regarding the collection of PROM data. Though considered vital for measurement of outcomes deemed important by patients, the extent to which PROM data were collected and its quality varied between centres. Professionals based in Worcester (small non-academic centre), Middlesbrough (medium-sized acute trust) and Leeds (large academic centre) were conscious that they did not collect PROM data post-treatment (although this did form an ongoing programme of work at Leeds). In comparison, the collection of PROM data at The Christie (large academic centre) appeared well established. Meanwhile, at the time of this study, a new electronic patient record platform called Connect was being readied for launch at Royal Marsden (large academic centre) with an aim to improve patient engagement that included the ability to interact with clinicians and contribute to care records.

From participant discussion, it appeared that larger centres viewed the analysis of data already collected as a key challenge, while both collection and analysis of key data presented challenges for the smaller and medium centres participating in the study. Nonetheless, as RWD encompasses multiple varieties of data and is key to evaluate outcomes in the rapid-learning approach, all participants were conscious of how data quality may affect the robustness of rapid-learning in practice.

### Theme 3: the maturity of data infrastructures and capacity for rapid-learning

This third theme describes the role of data infrastructures to address identified challenges regarding data quality and the capacity of centres to implement rapid-learning. Participants based in large centres roundly stressed the importance of integrating clinical and digital services to address data handling challenges and improve data interoperability. Digital learning and treatment planning personnel expressed the importance of greater data standardisation and accessibility, which they felt could be achieved through developing informatic infrastructures. Participants recognised that this would require time, investment and organisational commitment. Further potential benefits of more mature data systems, as highlighted by participants, included the collection of more granular detail and achieving greater data linkage and sharing between departments and within organisational boundaries. The following mantra—‘availability of the right data at the right time’—was repeated on several occasions by participants in response to often negative experiences of using complex data platforms.

Reflecting on the development needed to address data quality challenges, significant consideration was given by participants over the capacity of different cancer centres to implement rapid-learning. Identified set-up differences between cancer centres that participants believed could impact implementation included staff turnover, varying clinical interest and experience, difference in quality control mechanisms and capacity to build local evidence bases. Professionals based in Worcester (small non-academic centre) and Middlesbrough (medium-sized acute trust) respectively expressed some caution over their ability to implement rapid-learning citing lack of digital infrastructure, research time and space, and resources (therefore unable to build local evidence bases), which, in their view, were more commonly visible within larger academic centres. This said professionals based in larger cancer centres described how introducing change was not necessarily straightforward in their centres either, citing factors such as complexity of departments, established methods of practice and high patient turnover.

Where difficult to build a local evidence base (as expressed by professionals from Worcester and Middlesbrough), it was expected that some steer from organisations such as The Royal College of Radiologists or similar national-level guidance would be required to inform and support plans for implementation. In this respect, participants felt it would be more realistic for smaller and medium cancer centres to be part of wider organised implementation efforts. The need for greater data linkage and sharing between organisations was further emphasised here given the variable capacity to collect and analyse data in certain cancer centres. While it was acknowledged that individual centres face respective challenges, a consistent message across all interviews was that it was important to avoid rapid-learning becoming an ‘ivory-tower concept’ that restricted development to the host site (The Christie) and similarly well-connected, large centres.

### Theme 4: further support, education and evidence needed to convince stakeholders to adopt rapid-learning approaches

This fourth theme describes participants’ views towards what they considered may be needed to inform further acceptability of rapid-learning implementation across radiotherapy teams. A key methodological area that clinical professionals believed needed further clarity concerned the timeliness of learning cycles in rapid-learning. Progressing learning between rapid-learning cycles (and finding the optimal dose limit to the base of the heart in the RAPID-RT study) is contingent on measurement of acceptable toxicity.^18^ However, clinical professionals noted how toxicity may take time to develop, thereby potentially impacting the length of learning cycles. Subsequently, they questioned to what extent rapid-learning matched its moniker of ‘rapid’—‘how rapid is ‘rapid’?’ Furthermore, clinical oncologists described how cardiac risk is compounded by numerous factors^36 37^ but these data may not be routinely available or collected.^38 39^ Therefore, questions were raised over whether additional outcomes and measures would need to be collected as to provide more accurate toxicity data, and also how this would then impact existing routines of work. Further clarification of safety procedures in rapid-learning was also considered essential to secure ‘buy-in’ from professionals. This included, for example, clear information regarding critical data points and toxicity biomarkers, along with the processes through which trade-offs and unintended consequences would be managed. It was suggested that this could help convince radiotherapy teams of the ‘soundness’ of rapid-learning and increase confidence to link changes implemented with resultant outcomes observed.

To further address potential implementation challenges, participants noted how it would be advantageous to have dedicated channels of analytical support, particularly for data-related issues, along with more time and space for research exploration. Education for teams (particularly non-research staff) was widely acknowledged as rapid-learning is not based on standard competence and would require time and planning for familiarisation. In terms of the evidence required to change practice, proof of publication and peer review were considered minimum evidence (a practical demonstration of benefits) supported by professional debate (eg, through societies like The British Thoracic Oncology Group) with information and education dispersed nationally to different centres.

## Discussion

The rapid-learning approach was viewed favourably by different radiotherapy professionals across centres with potential to address current gaps in providing evidence supporting the development of radiotherapy. That includes, for example, receipt of timelier evidence and the possibility to offer more personalised treatment to patients. Tailored cancer treatment is seen as an unmet need within radiotherapy, to ensure that the individual patient receives optimal treatment and to optimise resources.^9 29 40^ Such potential benefits of rapid-learning contrast the well-documented challenges of RCT evidence, although it is important to note that all participants viewed rapid-learning as a complementary method to RCTs rather than replacement; this reflects the widely held consensus.^9–15^ Abernethy *et al* 15 emphasise the importance of carefully matching study design—whether using conventional RCTs, pragmatic rapid-learning or variations of both—to the complexity of the research question, considering that different outcomes are appropriate to different changes in practice. RCTs identify specific improvements, often clinical, in results between treatments in well-defined, controlled settings with a focus on homogeneity. In contrast, rapid-learning has a heterogeneous focus, measuring a wide spread of outcomes to evaluate intervention effectiveness in the routine setting.^6 9 15^ Price *et al* 1 describe how a rapid-learning model that uses a before-and-after design (comparing the outcomes of patients treated with the old-standard of care with patients treated with the modified technique) mirrors the current clinically accepted model for incrementally updating radiotherapy practice. However, there is potential for bias or uncontrolled confounding which must be accounted for in this design.^1 9 15^

The quality of data and informatic infrastructures appear a key obstacle to navigate for potential implementation across centres; this is not a new finding.^24–27^ Cancer centres shared their respective challenges of collecting, handling and analysing different datasets. Concerns around data are legitimate as rapid-learning requires access to, and analysis of large volumes of diverse data (particularly with respect to the treatments used, but also in terms of patient characteristics) if it is to enhance inclusivity and generalisability in the development of radiotherapy treatment.^24 26 28 41^ This said, Price *et al* 1 describe how the increasing prevalence of EHRs and growing number of large-scale multicentre learning health systems demonstrate that a lack of suitable informatics infrastructure is unlikely to be the main factor limiting the uptake of rapid-learning in the future.

The capacity of different centres to implement rapid-learning may be further influenced by their set-up corresponding to challenges presented by staff turnover, varying clinician interest and experience, and capability to build and develop local evidence bases. Subsequently, potential implementation of rapid-learning in smaller, non-academic settings was seen realistically as being part of wider organised implementation efforts, requiring the involvement of regulatory bodies and opinion formers to provide guidance. As suggested by several participants, there is responsibility on centres considered to have greater academic and resource capacity to lead on implementation efforts and share learning as to avoid rapid-learning becoming an ‘ivory-tower concept’. Articulating the short and long-term value of rapid-learning in practice, through initial ‘early-adopter’ sites, has been identified as a key facilitator for widespread adoption of rapid-learning across centres.^42 43^ Finally, participants identified the need for further clarification in areas such as method safety to increase clinical confidence and improve stakeholder buy-in for implementation. This is perhaps unsurprising as there appears to be a reported lack of practical guidance regarding the implementation of rapid-learning in radiotherapy departments.^1 15^

### Implications for research and practice

On review of the study findings, we suggest the following implications for research and practice that may warrant further exploration. The need to collect additional baseline data or different toxicity measures may be important given that clinical professionals discussed how numerous factors impact cardiac toxicity but this data may not always be routinely collected. In the absence of such data, professionals may find it difficult to definitively attribute the changes introduced to the resultant outcomes observed. Similarly, then, study teams may need to clarify key clinical markers or conduct further research as to satisfy ethical and safety concerns, clear uncertainties and increase confidence in rapid-learning approaches.^44–46^

There was shared consensus among centres regarding the significant role of organisations in fostering learning cultures and creating appropriate conditions for implementation, for example, time, resource, funding, education and training.^43 47–49^ We suggest further exploration may be needed to review how rapid-learning implementation impacts staff workload and routines, for example, collection, reporting and analysis of data. Radiotherapy teams already encounter tension between immediate clinical service delivery and research time.^50 51^ Furthermore, extensive research reports strong correlation between clinician burn-out and workload demands associated with the utilisation and management of EHR data.^43 52^

This study will be proceeded by the next stage of the research which seeks to assess the practical experience of implementing rapid-learning in lung cancer (at The Christie).^30^ Interviews with radiotherapy professionals will be held towards the end of the first learning cycle with a focus on capturing experience of implementation and whether rapid-learning is meeting its initial expectations.

### Strengths and limitations

Key strengths of this research study are that it included the views and experiences of a diverse range of professionals based across different cancer centres which provided greater understanding of the distinct roles and processes involved when introducing a change to radiotherapy practice. The overwhelming majority of participants were knowledgeable about current processes involved in radiotherapy practice evaluation, the various forms of evidence informing practice and RWD. Subsequently, participants were able to use this knowledge to reflect on and compare with the rapid-learning approach.

Limitations include that more interviews took place with personnel based at The Christie compared with other centres. We acknowledge that there is risk for potential bias within the findings presented as views expressed by participants may be overwhelmingly representative of a singular centre. We have explained the reasons for conducting more interviews at The Christie. The is the first site at which implementation of rapid-learning in lung cancer radiotherapy takes place and we further anticipated that some participants may be more familiar with the rapid-learning concept, thereby holding informed views that we intended to capture. It is further possible that some participants interviewed, across centres, may hold prior interest in the topic area, and therefore, agreed to participate in the study. Given rapid-learning had not been implemented at any of the sites at the time of this study, participants’ responses could be best categorised as hypothetical rather than based in practical experience. As a result, we stress that the findings presented in this article are participants’ views and the authors’ interpretation of them.

## Conclusion

Rapid-learning approaches may offer timely alternatives to traditional clinical trial studies for the evaluation of changes in radiotherapy, thereby increasing their acceptability for practice. However, as identified by different professional groups, there are several factors that may impact the feasibility of rapid-learning implementation. Access to high-quality data remains a concern across centres, while the variable capacity of different centres to develop appropriate settings required for implementation also poses a challenge. To this effect, developing data and digital infrastructures to improve data interoperability and sharing of data across organisations, along with the provision of practical and educational support (eg, method clarification, analytical support, guidance from regulatory bodies and resource investment) were identified as crucial steps for implementation. Such steps are likely to improve clinician confidence and strengthen the evidence base needed to support the implementation of rapid-learning in practice.

## Data Availability

Data are available on reasonable request.
