# Supplementary material for: Feasibility of implementing a rapid-learning methodology to inform radiotherapy treatments: key professional stakeholders’ views
Source: BMJ Oncol. 2024 Mar 13;3(1):e000226. doi: 10.1136/bmjonc-2023-000226 (PMC11235030; doi:10.1136/bmjonc-2023-000226)
Supplement: Supplementary data [file bmjonc-2023-000226supp001.pdf]

Supplementary File 1 – Interview schedules. Two interview schedules were designed and used to elicit key responses from participants informed by their respective roles and experiences.

**A) Interview Schedule**<br>(for clinic-based personnel e.g. clinical oncologists, physicists, treatment planning)
**Current Practice**
Q. Could you please describe a specific change to radiotherapy practice that you have experienced recently?
Q. What are the types of evidence you consult when making clinical decisions?
**Exploration of rapid-learning and real-world data (RWD)**
Q. Do you currently use routinely collected data about patients to inform any stage of the radiotherapy process?
Q. What is your understanding of real-world data and real-world evidence?
Q. What are your thoughts on the quality of data available to use?
Q. Is quality improvement (QI) or auditing something you are actively involved in or something that is visible within the area that you work in?
Q. What is your understanding of rapid-learning methodology?
Introduction and discussion of rapid-learning approach – infographic attached as appendix within participant information sheets
**Change and expectation**
Q. What do you believe are the implications for day-to-day practice if you were expected to integrate RWD into decision-making?
Q. Would you be confident that RWD will provide enough or adequate evidence to change practice?
Q. Do you have any concerns of using rapid-learning methodology?
Q. What do you believe are the key factors that would need to be considered in the use of rapid-learning and RWD?
**Summary of ideas/closing comments**

**B) Interview Schedule**<br>(for management, data informatics and research personnel)
**Exploration of rapid-learning and real-world data (RWD)**
Q. What types of data are used to inform radiotherapy decisions?
Q. What is your understanding of real-world data and real-world evidence?
Q. How is the process of data sharing and linkage undertaken?
Q. What are your thoughts on the quality of patient data?
Q. How do governance measures influence preparation of data?
Q. Is quality improvement (QI) or auditing something you are actively involved in or something that is visible within the area that you work in?
Q. What is your understanding of rapid-learning methodology?
Introduction and discussion of rapid-learning approach – infographic attached as appendix within participant information sheets
**Change and expectation**
Q. What do you believe are the implications for day-to-day practice if you were expected to integrate RWD into decision-making?
Q. Would you be confident that RWD will provide enough or adequate evidence to change practice?
Q. Do you have any concerns of using rapid-learning methodology?
Q. What do you believe are the key factors that would need to be considered in the use of rapid-learning and RWD?
**Summary of ideas/closing comments**
