# Supplementary figures and images for: Feasibility of implementing a rapid-learning methodology to inform radiotherapy treatments: key professional stakeholders’ views
Source: BMJ Oncol. 2024 Mar 13;3(1):e000226. doi: 10.1136/bmjonc-2023-000226 (PMC11235030; doi:10.1136/bmjonc-2023-000226)

Supplementary File 2: Thematic coding structure

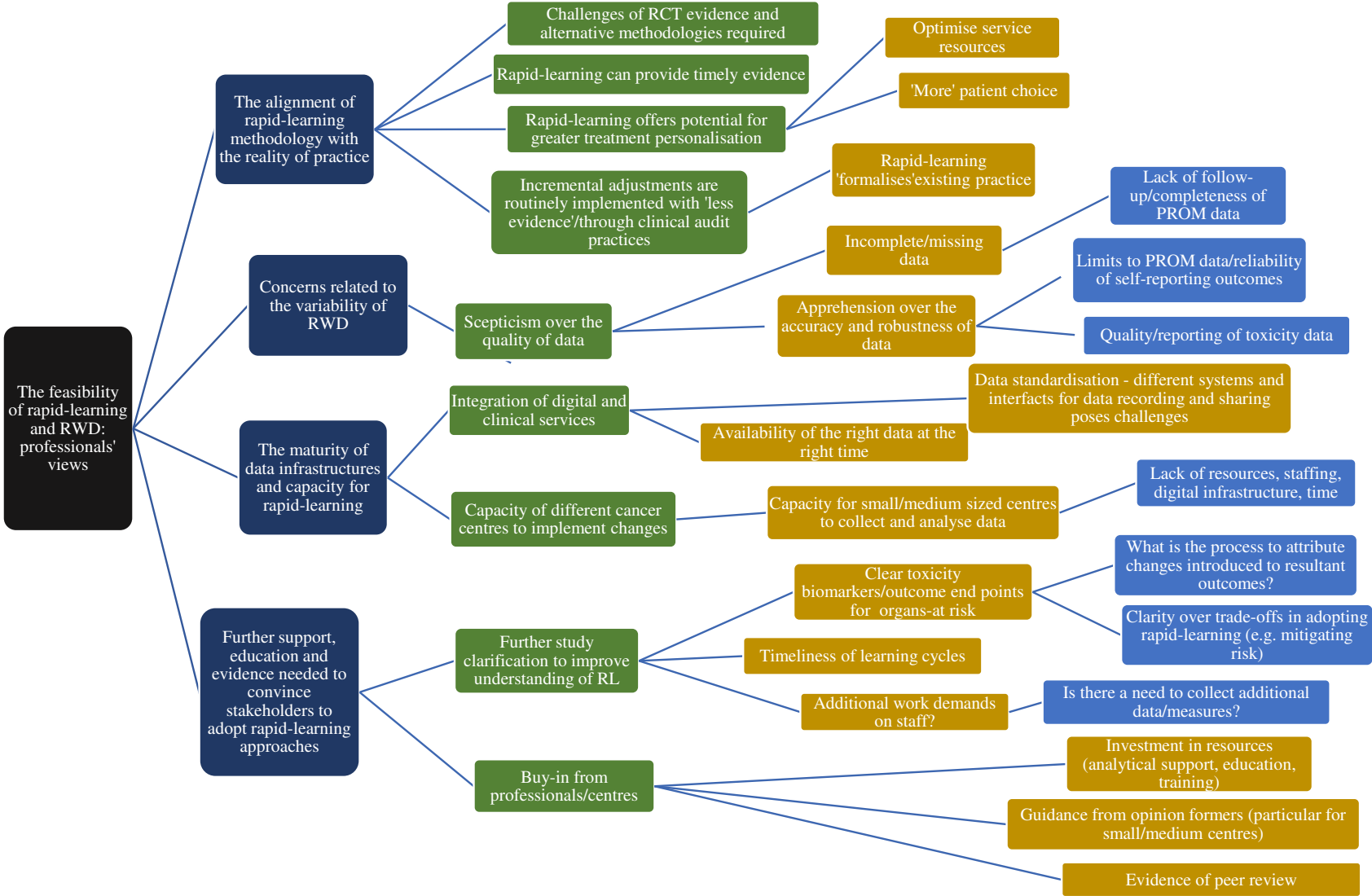

Supplement: Supplementary data [file bmjonc-2023-000226supp002.pdf]
